# Ultra-acute increase in blood glucose during prehospital phase is associated with worse short-term and long-term survival in ST-elevation myocardial infarction

**DOI:** 10.1186/1757-7241-22-30

**Published:** 2014-05-01

**Authors:** Hanna Vihonen, Ilkka Tierala, Markku Kuisma, Jyrki Puolakka, Jukka Westerbacka, Jouni Nurmi

**Affiliations:** 1 Department of Anaesthesia and Intensive Care, Helsinki University Central Hospital, P.O. Box 340, Helsinki, HUS, 00029, Finland; 2 Heart and Lung Centre, Helsinki University Central Hospital, Helsinki, Finland; 3 Helsinki EMS, Department of Anaesthesia and Intensive Care, Helsinki University Central Hospital, Helsinki, Finland; 4 Department of Medicine, Helsinki University Central Hospital, Helsinki, Finland

**Keywords:** Glucose, Myocardial infarction, Prehospital

## Abstract

**Background:**

The current study was to investigate the blood glucose changes in ultra-acute phase in patients with ST-elevation myocardial infarction (STEMI) and its associations with patient outcome.

**Methods:**

This study was a retrospective population-based observational study utilizing prospectively collected registry data complemented with laboratory data. All adult patients with STEMI treated by emergency medical services (EMS) in the city of Helsinki from January 2006 to December 2010 were included in the study. Both prehospital and hospital admission glucose values were available from 152 (32%) of all STEMI patients (n = 469).

**Results:**

Change in blood glucose from prehospital phase to emergency department admission was significantly higher in non-survivors within 30 days compared to survivors (+1.2 ± 5.1 vs. -0.3 ± 2.4 mmol/l [mean ± SD], P = 0.03). Furthermore, the 3-year survival rate was significantly lower in patients with an evident (≥2 mmol/l) rise in blood glucose (P = 0.02). In patients with impaired left ventricle function (best ejection fraction < 40%), blood glucose increased more compared to patients without it (1.2 ± 2.9 vs. 0.4 ± 2.7 mmol/l, P = 0.01). Increase in glucose was correlated with peak myocardial creatinine kinase (r = 0.17, P = 0.04) as a marker of increased size of infarct, but not with glycosylated haemoglobin A1C as a marker of chronic hyperglycaemia (r = −0.12, P = 0.27).

**Conclusions:**

In patients with STEMI, ultra-acute hyperglycaemia during prehospital phase is associated with increased mortality, impaired cardiac function and increased size of infarct.

## Background

Hyperglycaemia characterizes up to 50% of patients admitted with ST-elevation myocardial infarct (STEMI). Of those, 20-25% have previously diagnosed diabetes [1]. The non-diabetic patients may have stress-induced hyperglycaemia due to increased levels of insulin counter-regulating mediators such as proinflammatory cytokines, epinephrine, cortisol and tumour necrosis factor -alpha [2]. On the other hand of hyperglycaemic patients without previous diabetes at the time of STEMI, up to 40% actually were found having impaired glucose intolerance and 25% having diabetes three months after discharge [3]. Hyperglycaemia at admission in STEMI patients, regardless of having diabetes or not, is an independent predictor of in-hospital and long-term adverse outcomes like heart failure, cardiogenic shock and death [4-6]. For every 1 mmol/l increase in blood glucose level, there is a 4% increase in mortality in non-diabetic subjects and 5% increase in diabetic subjects [2]. Glucose levels of STEMI patients before arriving to hospital have not been described.

Whether treatment of hyperglycaemia improves outcome has not yet been definitely proved. The interventions to modify glucose metabolism have been initiated in most studies relatively late. For example, in the DIGAMI-II treatment was started 13 hours after first symptoms of acute myocardial infarction (AMI) and normoglycaemia was not achieved [7]. Recently, early management of dysglycaemia in acute myocardial ischemia has been studied. Selker et al. have shown prehospital glucose-insulin-potassium to decrease hospital mortality and cardiac arrest incidence of patients with acute coronary syndrome [8]. We have previously demonstrated that hyperglycaemia of stroke patients can be effectively treated in the prehospital phase by paramedics [9]. We also have demonstrated in patients resuscitated from out-of-hospital ventricular fibrillation that early hyperglycaemic response develops during prehospital phase to patients with poor prognosis [10].

As ultra-acute hyperglycaemia is associated with poor prognosis in other conditions involving pathophysiology of ischemia and reperfusion and as being a relatively easily modifiable factor, we aimed to describe the blood glucose changes in prehospital phase in patients with STEMI and association of these glucose changes with outcome of these patients.

## Methods

### Study design

This study was a retrospective population-based observational study utilizing prospectively collected registry data complemented with laboratory data. The study protocol was approved by Helsinki University Hospital institutional review board. According to local legislation, patient consent was not required as only patient records and registry data were used.

### Patients

We included all adult (over 18 years) patients with STEMI treated by emergency medical services (EMS) in the city of Helsinki (population of approximately 568 000) from January 1^st^ 2006 to December 31^st^ 2010. Only patients who were confirmed to have STEMI in the hospital were included. We excluded patients with missing prehospital and/or hospital admission glucose values.

### Treatment

In the city of Helsinki, EMS transports all the STEMI patients to the single university teaching hospital. Early presenters (<3 h from pain-onset) without cardiogenic shock or significant heart failure are primarily treated with prehospital fibrinolysis (tenecteplase) and all others with primary-PCI. All patients were vigorously evaluated for fibrinolysis-failure and early angiography is actively performed. Criteria for successful fibrinolysis are 50% resolution of ST-elevation and patient being painless and stabile in a time interval of 60–90 minutes. Rescue-PCI rate is 40%. Patients with successful fibrinolysis undergo angiography within 24 hours.

### Setting and data sources

The Helsinki EMS STEMI registry is a prospectively collected and includes all patients treated by EMS for STEMI. Emergency physician or medical supervisor is responsible of fulfilling the data into a structured data collection form after each case. Designated emergency physician verifies data based on EMS patient reports and hospital patient records.

Blood glucose was measured by EMS providers using the Optimum Xceed glucometer and MediSense Optimum electrodes (Abbott Laboratories, Alameda, CA). In these electrodes, the measurement is based on glucose dehydrogenase reaction and the glucometer is plasma calibrated. Prehospital blood glucose values were measured by ambulance personnel before arrival to hospital. Blood glucose measurement in the patients with chest pain is not mandatory in the standard operating procedure and thus depends on the consideration by the provider. Admission glucose values were included if taken within 24 hours of admission to hospital. Admission glucose was measured in central laboratory using hexokinase method in autoanalyser (Roche Diagnostics Hitachi 917, Hitachi Ltd., Tokyo, Japan). Prehospital glucose values were collected from the EMS patient reports. Admission glucose values and glycosylated haemoglobin A1c (HbA_1c_, closest value measured within one year of STEMI) values were obtained from electronic laboratory database. Cardiac infarct marker myocardial creatininekinase (CK-MBm) was obtained from electronic laboratory database. The left ventricle ejection fraction was measured by a cardiologist using an echocardiography and four-chamber planimetry. Data of co-morbidities of patients (coronary syndrome, hypertension, diabetes) were obtained from STEMI registry. All other data were collected from hospital electronic patient records. Patients were followed up to three years to assess mortality using national population register data.

### Analysis

The main outcome was 30-day survival and secondary outcomes impaired function (left ventricle ejection fraction <40%), ST-resolution, major bleeding and heart failure. Change in blood glucose during prehospital phase to hospital admission was compared between outcome groups. Correlations of change in glucose with HbA_1c_ and CK-MBm were calculated. Long-term survival up to three years was compared with subgroup with evident, over 2 mmol/l, ultra-acute rise in blood glucose. This cut-off value was chosen because of inaccuracy of glucometers might cause erroneously smaller rise in glucose comparisons. Additionally, we have previously shown it is possible to modify blood glucose level equivalently during prehospital phase [9].

Categorical data was analysed using either Fisher’s exact (two groups) test or chi-square test (more than two groups). Normally distributed data are reported as mean ± standard deviation (SD) and skewed variables as median and inter-quartile range (IQR). Spearman’s correlation was calculated for change in blood glucose and other continuing variables. Long-term survival was analysed using Log-rank (Mantel-Cox) test. All statistical analyses were carried out using GraphPad Prism 5.0 for Mac OS X (GraphPad Software, San Diego, CA).

## Results

During the study period, the Helsinki EMS treated total of 469 patients with STEMI. Both prehospital and admission glucose values were available on one third of patients who were consequently included in the study (Table 1). Comparison of included and excluded patients is presented in Table 2, showing higher prevalence of diabetes and anterior STEMI within included patients. There was no difference in hemodynamic values between included and excluded patient groups. Median interval from onset of symptom to measurement of prehospital blood glucose was 65 (IQR 41 to 130) minutes, and to measurement of blood glucose after admission 170 (IQR 125 to 270) minutes.

**Table 1:** Available blood glucose values in prehospital and admission phase of all STEMI patients treated during the study period (n = 469)

		**Admission glucose measurement available**	
Prehospital glucose measurement available		Yes	No
	Yes	152 (32%)	6 (1%)
	No	287 (62%)	24 (5%)

Patients with both prehospital and admission values available were included in the study.

**Table 2:** Characteristics of patients with both prehospital and hospital admission glucose values available (included patients) and patients with missing glucose values (excluded patients)

	**All N = 469**	**Included patients N = 152**	**Excluded patients N = 317**	**P value**
Age (years), median, IQR	62, 55-72	63, 54-73	61, 55-72	0.41
Sex, male (%)	336 (72)	106 (70)	230 (73)	0.58
Hypertension, %	182 (39)	66 (43)	116 (37)	0.16
Coronary artery disease, %	101 (22)	37 (24)	64 (20)	0.15
Diabetes, %	63 (13)	38 (25)	25 (7.9)	<0.0001
Weight (kg), median, IQR	75, 60-86	80, 70-90	80, 70-90	0.75
Admission glucose (mmol/l), median, IQR	7.8, 6.6-9.2	7.8, 6.4-9.8	7.7, 6.7-9.1	0.79
Localization of infarct, anterior (%)	235 (50)	71 (47)	164 (52)	0.03
Glycosylated haemoglobin A1_C_ (%)*, median, IQR/Glycosylated haemoglobin A1c (mmol/l), median IQR	5.9, 5.6-6.4/41, 38-46	6.0, 5.7-6.6/42, 39-49	5.8, 5.5-6.3/40, 37-45	0.02/0.02
Systolic blood pressure (mmHg), mean ± SD	142 ± 34	141 ± 33	143 ± 34	0.72
Pulse rate (beats per minute), mean ± SD	76 ± 22	75 ± 20	77 ± 22	0.34
Reperfusion strategy				
Thrombolysis	307 (65)	110 (72)	197 (62)	0.08
Primary PCI	124 (26)	31 (20)	93 (29)	
No reperfusion therapy	38 (8.1)	11 (7.2)	27 (8.5)	

IQR, interquartile range; SD, standard deviation; PCI, percutaneous coronary intervention.

*Available in 89 and 144 cases of included and excluded patients, respectively.

**Figure 1:**
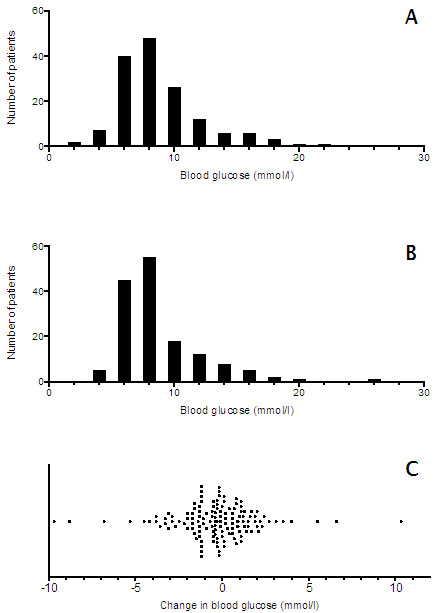
**Distribution of blood glucose values in prehospital (panel A), admission (panel B) and ultra-acute change of prehospital and admission (panel C).** Every dot represents a single patient.

Overall, no significant difference was observed between prehospital and admission glucose values (8.8 ± 3.3 mmol/l and 8.7 ± 3.4 mmol/l, respectively, P = 0.50). Change in glucose during prehospital phase in all patients was −0.3 ± 2.4 mmol/l. This change did not differ between 38 diabetic patients and 114 non-diabetic patients (0.2 ± 3.2 mmol/l and −0.3 ± 2.7 mmol/l, respectively, P = 0.37). Change during prehospital phase was not correlated with HbA_1C_ (r = −0.12, P = 0.27) or dose of intravenous metoprolol (r = 0.12, P = 0.14). Distributions of prehospital and admission glucose values, as well as change of blood glucose, are shown in Figure 1.

Seventeen patients died within 30 days after admission. Increasing ultra-acute change during prehospital phase was significantly associated with 30-day mortality and impaired left ventricle ejection fraction (Table 3). Furthermore, change in glucose from prehospital to admission values was correlated with peak CK-MBm (r = 0.17, P = 0.04).

**Table 3:** Association of outcome and blood glucose trend from prehospital phase to hospital admission

	**Number of patients**	**Change in glucose from prehospital to admission (mmol/l)**	**P value**
30 days survival			0.03
Yes	135	−0.3 ± 2.4	
No	17	1.2 ± 5.1	
Ejection fraction			0.01
≥40%	126	0.4 ± 2.7	
<40%	26	1.2 ± 2.9	
Resolution of ST elevation			0.92
Yes	104	−0.2 ± 2.5	
No	48	−0.1 ± 3.5	
Major bleeding			0.07
No	147	−0.2 ± 2.8	
Yes	5	2.1 ± 2.7	
Acute heart failure or cardiogenic shock			0.54
No	120	−0.2 ± 2.6	
Yes	32	0.1 ± 3.5	

**Figure 2:**
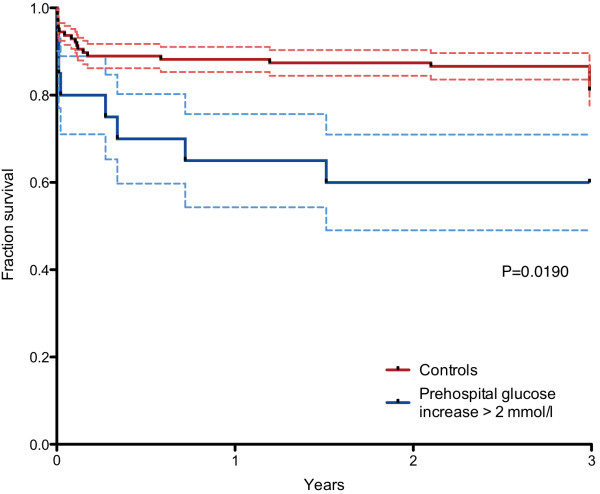
Survival curves of patients with and without ultra-acute over 2 mmol/l increase in blood glucose during pre-hospital phase.

Three-year survival was significantly lower in patients with evident, over 2 mmol/l, increase in blood glucose from prehospital phase to admission with a hazard ratio of 3.78 (95% CI 1.30 to 11.7, P = 0.02). The survival curves of these groups are presented in Figure 2.

## Discussion

This is the first study to demonstrate an association between ultra-acute increase of blood glucose during prehospital phase and mortality in STEMI patients. Patients with increasing blood glucose had higher short-term mortality. Interestingly, this outcome difference was remained in three-year long-term survival analysis. Early hyperglycaemic response seems to develop during prehospital phase and is more pronounced in patients with poor outcome. An ultra-acute increase in blood glucose was associated with decreased cardiac function and increase in marker of myocardial ischemia.

Previously it has been shown that hyperglycaemia at admission is associated with poor short-term outcome whereas chronic hyperglycaemia predicts long-term outcome in the patients with AMI [11]. Diabetes is well-known risk factor for cardiovascular events. Acute hyperglycaemia may be an independent factor that potentially reflects more severe condition of the patient or alternatively has harmful effect per se, and thus is associated with higher mortality to AMI. In the current study, ultra-acute blood glucose change during prehospital phase was not associated with diabetes or level of HbA_1C_, as an indicator of chronic hyperglycaemia. This implies that these are two distinct phenomena. Earlier studies on acute hyperglycaemia have focused to single blood glucose at admission. The current study implies that ultra-acute change may have a significant role as well, even to long-term survival.

There are many potential mechanisms linking ultra-acute hyperglycaemia to higher mortality and morbidity in acute STEMI. Stress-induced acute hyperglycaemia is caused by catecholamines, growth hormone, cortisol and cytokines. Acute increase in those counter regulatory hormones lead to excessive increase in hepatic glucose production and insulin resistance resulting in hyperglycaemia. Insulin resistance causes excessive free fatty acid production, which aggravates inflammation and hence worsens endothelial dysfunction together with hyperglycaemia [12]. Endothelial dysfunction caused by hyperglycaemia is characterized by suppression of flow-mediated vasodilatation. This is manifested as impaired microcirculation despite successful reperfusion [13]. Hyperglycaemia is associated with adverse effects on platelet function, fibrinolysis, coagulation and ischemic preconditioning of the heart. These directly damage the myocardium [14,15]. In the current observational study, hyperglycaemic response was associated with impaired myocardial function and greater infarct size although the definite mechanisms cannot be determined.

Whether glucose control using insulin is beneficial in STEMI is so far controversial. Major studies have found no outcome benefit of GIK infusion after STEMI [7,16]. However, GIK infusion does not lead rapid control of hyperglycaemia which has not been well studied yet. GIK has been preferred as insulin *per se* might have some beneficial effects in STEMI despite of glucose levels [17].

The main limitation of this study is that only one third of STEMI patients treated during the study period had sufficient data for analysis. This may result in a selection bias. In the study population diabetics and patients with anterior STEMI were overly presented. Data of potential previous heart failure was also absent. A retrospective design of the study limited also the study, although data collection was mainly based on prospectively collected registry data. The blood glucose values in the two time points were measured using different methods, which may have some effect on the results.

## Conclusions

This study demonstrated that in patients with STEMI, ultra-acute hyperglycaemic response developing during prehospital phase is associated with decreased cardiac function, increased size of infarct and increased 30-day mortality. Furthermore, also long-term survival is lower in patients with marked ultra-acute increase in blood glucose. These findings implicate need of further studies to evaluate results of modulation of this ultra-acute hyperglycaemia.

## Competing interests

The authors declare that they have no competing interests.

## Authors’ contributions

HV and JP obtained the data. HV and JN researched data. HV, JN and IT wrote the manuscript. JP, JW and MK reviewed and edited the manuscript. All authors read and approved the final manuscript.
